# Neuroinflammation and neurologic deficits in diabetes linked to brain accumulation of amylin

**DOI:** 10.1186/1750-1326-9-30

**Published:** 2014-08-22

**Authors:** Sarah Srodulski, Savita Sharma, Adam B Bachstetter, Jennifer M Brelsfoard, Conrado Pascual, Xinmin Simon Xie, Kathryn E Saatman, Linda J Van Eldik, Florin Despa

**Affiliations:** 1 Department of Pharmacology and Nutritional Sciences, University of Kentucky, Lexington, KY, USA; 2 Sanders-Brown Center on Aging, University of Kentucky, Lexington, KY, USA; 3 Spinal Cord and Brain Injury Research Center, University of Kentucky, Lexington, KY, USA; 4 AfaSci Research Laboratories, AfaSci, Inc., Redwood City, CA, USA; 5 Department of Physiology, University of Kentucky, Lexington, KY, USA; 6 Department of Anatomy and Neurobiology, University of Kentucky, Lexington, KY, USA

**Keywords:** Diabetes, Alzheimer’s Disease, Amylin, Pre-diabetes, Insulin Resistance, Inflammation, Behavior

## Abstract

**Background:**

We recently found that brain tissue from patients with type-2 diabetes (T2D) and cognitive impairment contains deposits of amylin, an amyloidogenic hormone synthesized and co-secreted with insulin by pancreatic β-cells. Amylin deposition is promoted by chronic hypersecretion of amylin (hyperamylinemia), which is common in humans with obesity or pre-diabetic insulin resistance. Human amylin oligomerizes quickly when oversecreted, which is toxic, induces inflammation in pancreatic islets and contributes to the development of T2D. Here, we tested the hypothesis that accumulation of oligomerized amylin affects brain function.

**Methods:**

In contrast to amylin from humans, rodent amylin is neither amyloidogenic nor cytotoxic. We exploited this fact by comparing rats overexpressing human amylin in the pancreas (HIP rats) with their littermate rats which express only wild-type (WT) non-amyloidogenic rodent amylin. Cage activity, rotarod and novel object recognition tests were performed on animals nine months of age or older. Amylin deposition in the brain was documented by immunohistochemistry, and western blot. We also measured neuroinflammation by immunohistochemistry, quantitative real-time PCR and cytokine protein levels.

**Results:**

Compared to WT rats, HIP rats show *i*) reduced exploratory drive, *ii*) impaired recognition memory and *iii*) no ability to improve the performance on the rotarod. The development of neurological deficits is associated with amylin accumulation in the brain. The level of oligomerized amylin in supernatant fractions and pellets from brain homogenates is almost double in HIP rats compared with WT littermates (P < 0.05). Large amylin deposits (>50 μm diameter) were also occasionally seen in HIP rat brains. Accumulation of oligomerized amylin alters the brain structure at the molecular level. Immunohistochemistry analysis with an ED1 antibody indicates possible activated microglia/macrophages which are clustering in areas positive for amylin infiltration. Multiple inflammatory markers are expressed in HIP rat brains as opposed to WT rats, confirming that amylin deposition in the brain induces a neuroinflammatory response.

**Conclusions:**

Hyperamylinemia promotes accumulation of oligomerized amylin in the brain leading to neurological deficits through an oligomerized amylin-mediated inflammatory response. Additional studies are needed to determine whether brain amylin accumulation may predispose to diabetic brain injury and cognitive decline.

## Background

Patients with type-2 diabetes (T2D) are at increased risk for developing cerebrovascular injury and cognitive decline [1-4]. Mechanisms implicated by prior work include atherosclerotic disease [1-4] and derangements in brain responsiveness to insulin [1-4], which are also common in “non-diabetic” patients (see Reference [1], for a recent review). We have recently [5] shown that brain tissue from patients with T2D and cerebrovascular dementia or Alzheimer’s disease (AD) contains significant accumulation of the pancreatic hormone amylin (islet amyloid polypeptide). In this paper, we report effects of amylin accumulation on brain function in an animal model.

Amylin, a 37 amino acid peptide with amyloidogenic properties, is synthesized and co-secreted with insulin by pancreatic β-cells [6] and plays a complex role in modulating peripheral energy balance. Some of the metabolic effects exerted by amylin are opposite those of insulin [7-10]. For example, amylin restrains insulin secretion from pancreatic β-cells [7] and reduces glycogen synthesis and glucose uptake in isolated muscle strips [8-10]. In addition to its role in peripheral metabolic processes, amylin exerts dual effects on the blood pressure by stimulating renal release of renin [11] and relaxation of blood vessels [12,13]. Amylin also crosses the blood–brain barrier [14] and is a potent inhibitor of ingestive behavior [15]. In accordance with amylin’s anorexic effects, amylin binding sites were detected in feeding-related centers, including the brainstem, hypothalamic nuclei and parabrachial area [7,15-17]. A dense distribution of high-affinity amylin binding sites was also identified in nucleus accumbens [18]. Direct infusion of amylin into nucleus accumbens diminished exploration and appetitive approach behaviors [15]. A recent study [19] reported that intraperitoneal injection of amylin reduced the amyloid burden in a mouse model of AD. The authors [19] suggest that the amylin hormone mediates the translocation of β-amyloid (Aβ) peptide from the brain.

Human amylin hormone may lose its function through oligomerization and amyloid formation, which can be toxic if it accumulates in tissues. For example, the majority of patients with T2D have abundant amylin amyloid deposition in the pancreas [20-22]. Pancreatic islets positive for amylin amyloid show elevated β-cell apoptosis and decreased β-cell area suggesting a role of amylin amyloid formation in the development of T2D [23]. Amylin amyloid cytotoxicity was also demonstrated *in vitro*[24-29] and in animal models of islet amyloid formation [30-33]. Apparently, the most toxic species of amyloid are not the large interstitially formed plaques, but the small oligomers [34] which can infiltrate cellular membranes [24] leading to Ca^2+^ dysregulation [24,31], oxidative stress [27,28] and inflammation [29]. Likely causes of amylin oligomerization may include *i*) amyloidogenicity [7] and *ii*) hypersecretion [7,20-22,31]. Hypersecretion of amylin, i.e. hyperamylinemia, coincides with hyperinsulinemia [6,7] and is common in individuals with obesity or pre-diabetic insulin resistance [7,35,36]. Human amylin overexpressed (3-fold) in the pancreas of rodents leads to amylin oligomerization, amyloid formation, β-cell apoptosis and development of T2D [30,31]. We recently showed that a “human” hyperamylinemia may accelerate the development of diabetic heart disease [37] and trigger arrhythmia [38] in an animal model of myocardial amylin accumulation.

Accumulation of oligomerized amylin (not necessarily amyloid) was also detected in kidneys [39], heart [37], and brain [5] tissue from diabetic patients, suggesting that hyperamylinemia may induce systemic effects in humans. In the brain, oligomerized amylin deposits and amylin amyloid were identified within blood vessel walls, perivascular space and tissue parenchyma [5]. Distribution of amylin pathology is similar (and complementary) to that of Aβ [5]. Moreover, amylin interacts directly with Aβ forming mixed amylin-Aβ oligomers [5]. We, therefore, hypothesize that hyperamylinemia may promote amylin oligomerization and accumulation of oligomerized amylin in the brain, which may constitute a pathological substrate for diabetic brain injury and cognitive decline.

Relevant to this novel hypothesis, rodent amylin does not form amyloid [40] due to proline substitutions at positions 25, 28, and 29 and is not cytotoxic [31]. Overexpression of wild-type (WT) rodent amylin in mice does not induce amyloid deposition, β-cell loss or T2D [31]. We exploit this fact by using a rodent model expressing human amylin in the pancreas to assess mechanistically the impact of a “human” hyperamylinemia on brain function.

The aims of the present study were twofold. First, we tested whether rats overexpressing human amylin in the pancreas show brain dysfunction associated with amylin accumulation in the brain. Secondly, we investigated possible mechanisms underlying the effects of amylin accumulation on brain function.

## Results and discussion

**Figure 1:**
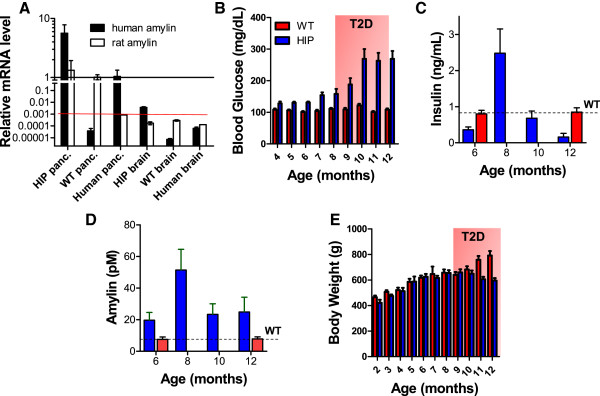
**The HIP rat: a “humanized” animal model of hyperamylinemia. A** – Specificity of human amylin RNA expression in the pancreas in HIP rats documented by qRT-PCR. Human pancreas was used as positive control for human amylin mRNA, while WT rat brain and pancreas were negative controls. Human amylin mRNA in the HIP rat brain is below the limit of detection (red line indicating that the signal measured in the qRT-PCR experiment was greater than 35 CT). Note also the logarithmic scale and scale break. **B** – Blood glucose in HIP rats and WT littermates. In HIP rats, the non-fasted blood glucose increases in the 150–200 mg/dl range (pre-diabetes) by ~7-9 months of age. By 10–12 months of age, the non-fasted blood glucose rises to over 200 mg/dl and the HIP rats develop T2D. (N = 32 HIP rats, N = 15 WT littermates). **C** – Blood insulin in HIP rats and WT littermates. In pre-diabetic HIP rats (7–9 months old), pancreatic β-cells compensate for insulin resistance by increasing secretion of insulin (hyperinsulinemia). (N = 32 HIP rats, N = 15 WT littermates). **D** – Blood amylin in HIP rats and WT littermates. Parallel with hyperinsulinemia (C), HIP rats develop hyperamylinemia by 7–9 months of age. (N = 32 HIP rats, N = 15 WT littermates). **E** – Body weight in HIP rats and WT littermates. HIP rats and WT littermates have similar body weights before the onset of T2D. With the development of T2D, HIP rats lose weight. (N = 32 HIP rats, N = 15 WT littermates).

To assess the impact of a “human” hyperamylinemia on brain structure and function, we used a rat model which overexpresses human amylin in the pancreas (the HIP rat) [30]. We documented the specificity of human amylin RNA expression in the pancreas in HIP rats by quantitative real-time PCR (qRT-PCR). The human pancreas served as positive control for detecting the presence of human amylin gene in HIP rats. In Figure 1A, we see that the mRNA level of human amylin in the HIP rat pancreas is comparable to that in human pancreas. Also, because human amylin gene should not be present in WT rat tissues, pancreas and brain protein homogenates from WT rats were used as negative controls. Indeed, the signal of human amylin mRNA in WT rat tissues is below the limit of detection (Figure 1A; red line). Present results (Figure 1A) also show that the human amylin mRNA in the HIP rat brain is within the limit of detection. We, therefore, interpret the lack of amylin expression in the HIP rat brain as conclusive support for using the HIP rat model to test the hypothesis that brain amylin deposition is from pancreatic β-cells.

Prior studies using the HIP rat model demonstrated that overexpression of human amylin promotes amylin oligomerization and deposition in pancreas [30] and extra-pancreatic tissue [37]. Amylin oligomerization and deposition in the pancreas leads to a gradual decline of β-cell mass, increase of non-fasted blood glucose in the 150–200 mg/dl range (Figure 1B) by ~7-9 months of age (pre-diabetes) and onset of full-blown T2D by 10–12 months of age (non-fasted blood glucose >200 mg/dl; Figure 1B). Insulin and amylin secretion is maximum at ~8 months of age (Figure 1C and Figure 1D), followed by a significant decline of blood insulin and amylin with the development of T2D (non-fasted blood glucose >200 mg/dl). HIP rats included in the present study were 8 months of age or older and showed hyperinsulinemia and hyperamylinemia at the time they were investigated. WT littermate rats displayed no change in blood glucose, insulin and amylin levels (Figure 1B, Figure 1C and Figure 1D). Metabolic profiles of HIP rats and WT littermate rats were published elsewhere [30].

Considering the possible role of amylin in controlling satiety [7,17], it is interesting to note that HIP rats and WT littermates show no difference in body weight before onset of T2D (Figure 1E). With the development of T2D, HIP rats lose weight (Figure 1E), which is also common in humans with T2D [41]. The results suggest that chronic overexpression of human amylin may mitigate the amylin-mediated anorexic effect in rats.

### Chronic hyperamylinemia is associated with neurological deficits

**Figure 2:**
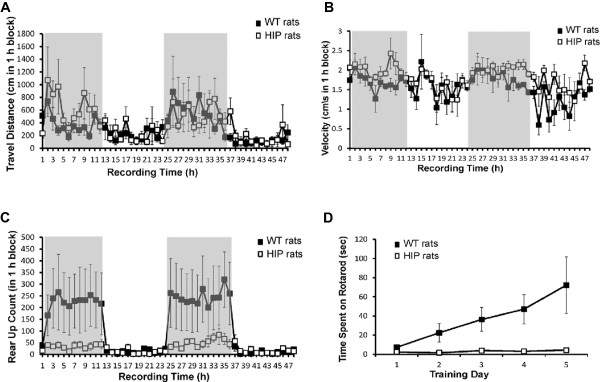
**Effects of hyperamylinemia on behavior.** The SmartCageTM system was used for automated analysis of spontaneous activity in HIP rats and WT rats. The activity time was measured as infrared photobeam breaks. Each rat was monitored in an ordinary home cage placed within the SmartCageTM platform for 72 hours, including the initial 24 hours for accommodation/ acclimation. After the initial 24 hours of acclimation, recordings were made for the next 48 hours. The recording time 1 started at 1 a.m. Grey area denotes nighttime. **A** – Total travel distance was similar in HIP rats and WT rats. **B** – HIP rats showed unchanged ambulatory velocity compared to WT rats. **C** – Compared to WT rats, HIP rats had decreased exploratory activity demonstrated by the low spontaneous rear up count. **D** – Vestibulomotor performance in HIP rats and WT rats was assessed by the rotarod performance test. Same animals as in above were subjected to the rotarod performance test at a rod-rotatxing speed increased from 0 rpm to 15 rpm within 1 min. HIP rats showed no ability to improve performance on the rotarod. In contrast, WT rats improved rotarod time with each day of training.

Subtle but significant changes in brain function are frequently observed in patients with T2D [1-4]. Specific disturbances include psychomotor speed and memory [1-4]. Such changes have been difficult to reproduce in rodent models [42]. Part of this problem arises from the fact that T2D does not spontaneously develop in rodents and is induced by genetic manipulations [30]. Depending on the transgene, some animal models show cognitive impairment even in the absence of hyperglycemia [42]. In other rodent models of T2D, such as the Zucker Diabetic Fatty (ZDF) rat, brain function remains intact [42], despite the development of essential features of T2D, including hyperglycemia, insulin resistance and hyperinsulinemia. The resistance of ZDF rats to diabetic brain injury and cognitive decline is attributed [42] to hyperinsulinemia, a condition believed to protect brain structure and function in T2D [43]. In addition to impaired glucose metabolism and hyperinsulinemia, the HIP rat bears a “human” hyperamylinemia, which could affect brain function. Here, we investigated a possible impact of hyperamylinemia on brain function using home cage activity monitoring, rotarod and novel object recognition protocols.Age-matched (~9 months old) WT rats and HIP rats were acclimated to the SmartCageTM system for a day before the recording time begun. The activity time was assessed by analyzing the infrared photobeam breaks. Based on the home cage activity monitoring, there were no significant differences in horizontal (x, y) activity. Total travel distance (Figure 2A) and velocity (Figure 2B) were similar in HIP rats and WT rats. The count of spontaneous rear up was lower in HIP rats compared to WT rats (Figure 2C) suggesting decreased exploratory activity in HIP rats. We further analyzed the vestibulomotor performance of HIP rats and WT rats by using the rotarod test. Animals in the same groups as in above were subjected to the rotarod performance test at a rod-rotating speed increased from 0 rpm to 15 rpm within 1 min. In the first day of training, HIP rats and WT rats had similar vestibulomotor performance (Figure 2D). However, HIP rats demonstrated no ability to improve performance on the rotarod (Figure 2D). In contrast, WT rats increased the rotarod time with each day of training.

Compared to age-matched WT rats, HIP rats show significantly reduced exploratory drive and impairment in the rotarod test. Diminution of exploratory drive in HIP rats is consistent with prior studies [15] demonstrating that intracerebroventricular infusion of amylin decreases ambulation and rearing in rats. Because HIP rats have preserved locomotion ability (Figure 2A,B), their poor rotarod test performance (Figure 2D) suggests impaired coordination or/and learning deficits. Additional studies are needed to clarify the link between brain amylin pathology and impaired coordination in the HIP rat model.

**Figure 3:**
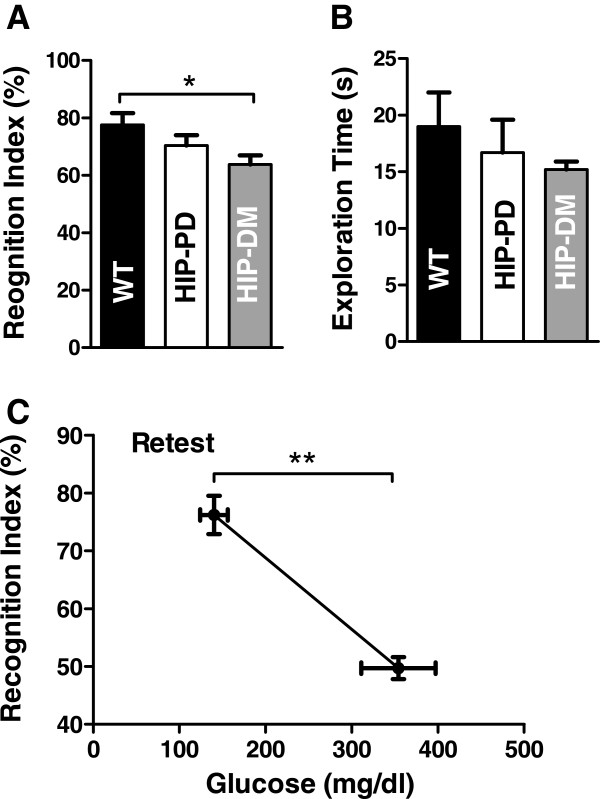
**Effects of hyperamylinemia on long term memory.** The Novel Object Recognition (NOR) protocol was used to assess possible changes in recognition memory in HIP rats. The recognition index (RI) is defined as the ratio of time spent exploring a novel object over the total time spent exploring both familiar and novel objects. **A** – In comparison with WT rats (N = 10), diabetic HIP (HIP-DM) rats (i.e., non-fasted blood glucose >200 mg/dl; N = 16) showed significant impairment in recognition memory (*P < 0.05). Pre-diabetic HIP rats (HIP-PD; non-fasted blood glucose <200 mg/dl; N = 10) were not different from either WT or HIP-DM rats. **B** – Total exploration times during the object recognition trial were not significantly different among wildtype (WT), pre-diabetic HIP, and diabetic HIP (HIP-DM) rats. **C** – A subset of pre-diabetic HIP rats (N = 3) was retested for the novel object recognition approximately 8 weeks after the initial trial, by which time they had developed full-blown T2D (i.e., blood glucose >200 mg/dl). Progressing to full diabetes led to a significant drop in the recognition memory in HIP rats (**P < 0.001) compared to the initial NOR test.

Hyperamylinemia may affect neuron function [44,45]. The Novel Object Recognition (NOR) protocol was used to assess possible changes in recognition memory in HIP rats versus age-matched WT rats. Both pre-diabetic HIP rats, (i.e., non-fasted blood glucose <200 mg/dl; rats ~9 months of age), and diabetic HIP rats, (i.e. non-fasted blood glucose >200 mg/dl; rats 10–12 months of age), were included in the study. The time spent exploring a familiar object and a novel object was recorded during a fixed duration trial. Diabetic HIP rats show a significant memory impairment, as indicated by reduced exploration of the novel object, as compared to WT rats (P = 0.0342; Figure 3A). Recognition memory function of pre-diabetic HIP rats was intermediate, but not significantly different from either WT rats or diabetic HIP rats (Figure 3A). Total exploration time during the recognition trial showed a similar pattern of decline in the HIP rats compared to WT rats, but the differences across groups were not statistically significant (Figure 3B). Since the recognition index is normalized by exploration time, lower exploration times should not influence cognitive scores in the NOR task. To assess possible regression in the recognition memory with the development of disease, a subset of animals with low blood glucose levels at the time of the initial NOR test were re-tested at a later time (~8 weeks), after they developed full-blown T2D (i.e., blood glucose >200 mg/dl). Progressing to full diabetes led to a significant drop in the recognition memory in HIP rats (P = 0.0022; Figure 3C). The decline in long-term recognition memory in HIP animals suggests a direct impact of hyperamylinemia on hippocampal neurons. In future experiments, we will test the integrity of synaptic function in hippocampal slices from HIP rats, which will help elucidating a possible link between hyperamylinemia and memory deficit in HIP rats.

The results suggest that chronic hyperamylinemia may ignite the development of neurological deficits, including changes in active/inactive rhythm, decreased exploratory activity and impaired vestibulomotor activity. Progressing to full-blown T2D appears to exacerbate hyperamylinemia-induced deleterious effects on brain function leading to learning and recognition memory deficits. Future studies should identify specific subtypes of behavior/cognitive domains affected in pre-diabetic HIP rats and fully-diabetic HIP rats.

### Chronic hyperamylinemia promotes accumulation of oligomerized amylin in the brain

**Figure 4:**
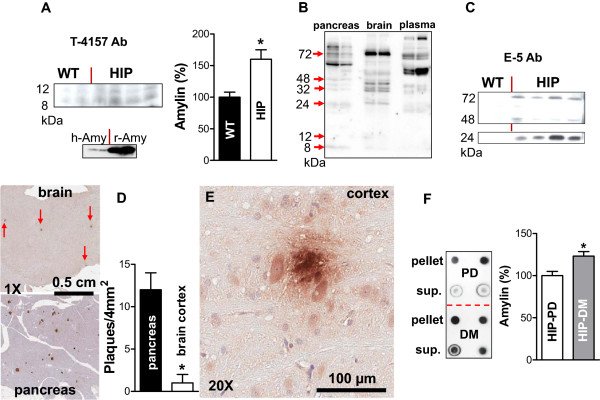
**Hyperamylinemia promotes amylin accumulation in the brain. A** – An anti-amylin antibody (T-4157) which binds both human amylin (h-Amy) and rat amylin (r-Amy), the latter with a higher avidity (bottom panel), detects amylin in brain protein homogenate from both WT rats and HIP rats. Total (rat and human) amylin content is about 50% greater in brain tissue from HIP rats. (*P < 0.05; N = 7 animals/group). **B** – Matched pancreas and brain supernatant samples and plasma from the same HIP rats (N = 2) were investigated for the presence of oligomerized amylin with the T-4157 anti-amylin antibody. **C** – An antibody specific for human amylin demonstrates that human amylin is present only in HIP brain tissue. Western blot analysis shows that amylin aggregates with various molecular weights accumulate in the brain. **D** – Amylin deposition within the brain in HIP rats was demonstrated by immunohistochemistry with the T-4157 anti-amylin antibody. Amylin immunoreactivity signal in pancreatic islets (bottom) is compared to that in brain tissue (top). The incidence of amylin plaque formation is more than ten times lower in the brain than in pancreatic islets, in HIP rats. (*P < 0.05; N = 3 animals). **E** – Large amylin deposits, i.e. ≥ 50 μm diameter are occasionally seen in brain parenchyma (cortex). **F** – Diabetic (DM) HIP rats show increased amylin accumulation in the brain compared to pre-diabetic (PD) HIP rats, which can be observed in both supernatant fraction (*P < 0.05) and pellets. For quantification of the amylin immunoreactivity signal, pellets of brain homogenates from HIP rats were treated with formic acid (overnight) and guanidine hydrochloride (for two hours) to disentangle larger amylin aggregates which may not bind to the anti-amylin antibody. (N = 4 animals/group).

The pancreatic β-cells in HIP rats express both human amylin and rat amylin (Figure 1A). The two peptides can form mixed amylin aggregates, as demonstrated by prior work [46,47]. Western blot analysis with an anti-amylin antibody which binds both human amylin and rat amylin (the latter, with a higher avidity; Figure 4A, bottom panel) shows that amylin accumulates in the brain of HIP rats. Small amylin aggregates, i.e. dimers (≅ 8 kDa) and trimmers (≅ 12 kDa), are also observed in supernatant fraction of brain homogenates from WT rats (Figure 4A). However, the amylin immunoreactivity signal is about 50% greater in brain tissue from HIP rats (Figure 4A). Matched samples of plasma and supernatant fractions of pancreas homogenate and brain homogenate from the same HIP rats were tested for the presence of oligomerized amylin by western blot (Figure 4B) with the same amylin antibody. The results suggest that oligomerized amylin is secreted from pancreatic islets in the blood (Figure 4B) and accumulates in the brain (Figure 4A). As expected, an antibody specific for human amylin demonstrates that human amylin is present only in HIP brain tissue (Figure 4C). Western blot analysis indicates the presence of oligomerized amylin at various molecular weights (MW), including ~24 kDa, ~48 kDa and ~72 kDa. However, an accurate estimation of human amylin content in HIP rat brains proves difficult to accomplish with currently available immunochemistry techniques.

Brain tissue from pre-diabetic HIP rats enrolled in the home cage activity study (Figure 2) was also analyzed by immunohistochemistry (Figure 4D and E) with the same anti-amylin antibody used in Figure 4A. The brain of HIP rats shows only modest amylin deposits (Figure 4D and E; brown). By comparing the amylin immunoreactivity signal in pancreatic islets (positive control; Figure 4D, bottom) versus brain tissue (Figure 4D, top), one can observe that the incidence of amylin plaque formation is more than ten times lower in the brain than in pancreatic islets, consistent with the amylin source in the pancreas. Large amylin deposits, i.e. > 50 μm diameter (Figure 4E), were occasionally seen in HIP rat brains. Compared to a common rodent model of AD [19], the density of proteinaceous deposits within the brain in HIP rats is significantly reduced. The low density of amylin plaques may be due to the fact that the HIP rat pancreas secretes both human amylin and rat amylin. Rodent amylin was shown to decrease the rate of amyloid formation by human amylin, *ex vivo*[46,47].Diabetic (DM; Figure 4F) HIP rats show increased amylin accumulation in the brain compared to pre-diabetic (PD; Figure 4F) HIP rats, which can be observed in both supernatant fraction (*P < 0.05) and pellets. For quantification of the amylin immunoreactivity signal, pellets of brain homogenates from HIP rats were treated with formic acid (overnight) and guanidine hydrochloride (for two hours) to disentangle larger amylin aggregates which may not bind to the anti-amylin antibody. Brain amylin level was higher in diabetic HIP rats due to a cumulative effect. This result also implies that blots exhibiting oligomerized amylin (Figure 4A) likely underestimate the amount of amylin in HIP rat brains.

### Accumulation of oligomerized amylin is associated with neuroinflammation

Accumulation of oligomerized amylin is a potent generator of inflammation in the pancreas [29]. We, therefore, propose the hypothesis that neurological deficits in HIP rats may involve an amylin-mediated inflammatory response in the brain.

**Figure 5:**
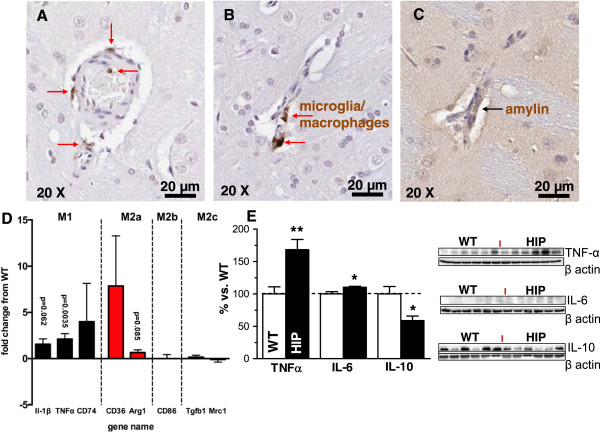
**Amylin deposition in the brain triggers an inflammatory response. A** and **B** – Immunohistochemistry analysis with an ED1 antibody on brain sections from HIP rats shows possible activated microglia/macrophages (arrows). **B** and **C** – Microglia/macrophages are particularly clustering around the small blood vessels in areas positive for amylin infiltration, as shown by serial staining with amylin **(C)** and ED1 **(B)** antibodies. **D** – qRT-PCR was used to analyze a subset of gene expression markers [48] defining microglia as classically activated (M1) or alternatively activated (M2_a/b/c_). The expression of both M1 and M2 phenotypic markers are increased in the cortex of HIP rats relative to WT rats suggesting that amylin deposition activates microglia. **E** – The association of brain amylin pathology with neuroinflammation was tested by western blots with TNF-α, IL-6 and IL-10 antibodies. Brain protein homogenates from HIP rats show elevated pro-inflammatory cytokines TNF-α and IL-6, while the anti-inflammatory cytokine IL-10 is down-regulated. Equal loading was verified by re-probing with a monoclonal anti-β actin antibody (*P < 0.05; **P < 0.01; N = 6 animals/group).

Immunohistochemistry analysis with an ED1 antibody on brain slices from HIP rats shows possible activated microglia/macrophages (red arrows, Figure 5A-B). Microglia/macrophages are particularly clustering around the small blood vessels in areas positive for amylin infiltration, as shown by serial staining with amylin (black arrow Figure 5C) and ED1 (Figure 5B) antibodies. To explore changes in microglia phenotype, we analyzed a subset of gene expression markers [48] defining microglia as classically activated (M1) or alternatively activated (M2_a/b/c_). The expression of both M1 and M2 phenotypic markers are increased in the cortex of HIP rats relative to WT rats (Figure 5D), suggesting that amylin deposition activates microglia. To confirm these changes at the protein level, we used TNF-α, IL-6 and IL-10 antibodies on western blot (Figure 5E). Supernatant of brain homogenates from HIP rats show elevated pro-inflammatory cytokines TNF-α and IL-6, while the anti-inflammatory cytokine IL-10 is down-regulated. These results suggest that amylin deposition in the brain triggers an inflammatory response, consistent with the pathological role of amylin in the pancreas [27-29].The results indicate that a “human” hyperamylinemia promotes accumulation of oligomerized amylin in the brain, which may trigger an inflammatory response leading to neurological deficits (Figures 2 and 3).

## Conclusions

**Figure 6:**
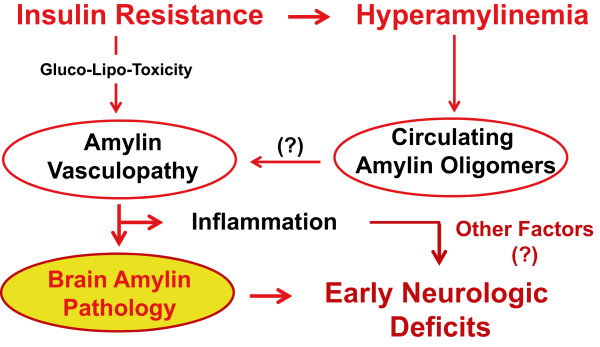
**Proposed pathological mechanism for the impact of hyperamylinemia on brain function**. Pre-diabetic insulin resistance triggers hyperamylinemia (which coincides with hyperinsulinemia) and leads to an increased secretion of oligomerized amylin from pancreatic islets in the blood. Circulating oligomerized amylin infiltrates the blood vessel wall causing blood–brain barrier injury and diffusion of oligomerized amylin in the brain, where it is toxic to the neuron. These pathological processes begin in pre-diabetes and may ignite the development of neurological deficits.

Using the HIP rat model, we showed that chronic hyperamylinemia promotes accumulation of oligomerized amylin in the brain which is associated with brain dysfunction. Our results indicate that these pathological processes begin in the pre-diabetic state and may ignite the development of neurological deficits, including changes in active/inactive rhythm, decreased exploratory activity and impaired vestibulomotor activity. Progressing to full-blown T2D appears to exacerbate oligomerized amylin-induced deleterious effects on brain function leading to recognition memory deficits. At the molecular level, our results suggest an amylin-mediated inflammatory mechanism. Finding of the activated microglia/macrophages lining small blood vessels in areas positive for amylin infiltration suggests a possible amylin-induced vasculopathy. Our proposed mechanism is displayed in Figure 6. Future studies are required to test the proposed mechanism and should focus on deciphering modulators of the amylin pathology in the brain, particularly the mechanism(s) by which human amylin infiltrates the brain. There is also a need to identify specific subtypes of behavior/cognitive domains affected in pre-diabetic HIP rats and fully-diabetic HIP rats. Possible validation from human studies may render the HIP rat as a useful animal model for pre-clinical therapeutic interventions to reduce the impact of diabetes on brain function.

The amylin hormone is known to induce anorexic effects through specific brainstem-localized receptors. Present results suggest that chronic overexpression of the human amylin hormone may mitigate the anorexic properties of this hormone. The only weight loss observed in HIP rats occurs after the development of T2D. Thus, the human amylin hormone may lose its function through hypersecretion and oligomerization, which is likely related to the amyloidogenic property of human amylin [40]. A similar pathological process may happen in humans with obesity or pre-diabetic insulin resistance who are susceptible to develop hyperamylinemia.

In summary, pathological changes in pancreatic islets could be linked with brain dysfunction through accumulation of oligomerized amylin. Brain amylin may constitute a pathological substrate for diabetic brain injury and cognitive decline. Blood amylin may be a novel potential therapeutic target for cerebrovascular injury and cognitive impairment.

## Methods

### Experimental animals

Animal studies received institutional approval. The HIP rat is a Sprague–Dawley (SD) rat that expresses human amylin in pancreatic β-cells (Figure 1A) and was developed as described in Ref. 30. We have recently [37,38] shown that the HIP rat is a clinically relevant animal model to study complications of T2D associated with amylin deposition in myocardium. WT littermates, which do not accumulate amylin in the pancreas and peripheral organs, served as controls. Only males were selected for this current study.

HIP rats were checked monthly for blood glucose levels (with a glucometer) and every other month for insulin and amylin levels (both, by ELISA, as previously [37] described). N = 32 HIP rats, N = 15 WT littermates and N = 10 SD rats were included in this study. WT littermates were matched for body weight with the HIP rats at the initial selection, when HIP rats were in pre-diabetes (non-fasted blood glucose <200 mg/dl. Based on retrospective data [37], our study was amply powered to assess the difference between amylin levels in the extrapancreatic tissue in HIP rats versus WT rats (P < 0.05).

### Statistical analysis

Data are expressed as mean±SEM. Statistical discriminations were performed using two-tailed unpaired Student’s t-test when comparing two groups, one-way analysis of variance (ANOVA) with the Bonferroni’s post-hoc test when comparing multiple groups and two-way ANOVA when comparing multiple groups for multiple conditions. P < 0.05 was considered significant.

### Quantitative real-time PCR (qRT-PCR)

Was used to test if human amylin is expressed in the HIP rat brain and to assess the amylin-induced inflammatory response in the HIP rat brain. Expression level of human amylin in pancreas and brain tissues from HIP rats and WT rats was assessed by quantitative reverse transcription real-time PCR (qRT-PCR) using a previously described method [5]. 18S ribosomal RNA (Applied Biosystems) was the endogenous control in the qRT-PCR experiment.

To assess possible gene expression levels associated with amylin-mediated inflammatory response in HIP rat brains, selected samples were assayed by the Applied Biosystems ViiA7 real-time PCR system, as we previously described [48]. A custom TaqMan array microfluidic card was used to analyze 8 samples in parallel with 48 different genes. The card contains a set of standard M1 markers (IL-1β, IL-6, TNFα, CD74, CD86, NO on) and M2 markers (Arg1, CD36, Mrc1, IL4-Rα, IGF1, IL-10, IL-1rn, TGFβ).

Gene expression analysis was performed as previously described [48]. Briefly, RNA was isolated using RNeasy mini-columns (Qiagen) with on-column DNase treatment (Qiagen) according to the manufacturer's protocol. RNA quantity and quality were determined using A260/A280 readings by NanoDrop (Thermo Scientific). Reverse transcription (RT) was done following the manufacturer's protocol using High Capacity cDNA Reverse Transcription Kit (Applied Biosystems). Real-time PCR was performed using the TaqMan Gene Expression assay kit (Applied Biosystems) according to the manufacturer's instructions on a ViiA™ 7 Real-Time PCR System (Applied Biosystems). The following TaqMan probes (Applied Biosystems) were used: Il-1β (Rn00580432_m1); TNFα (Rn01525859_g1); Cd74 (Rn00565062_m1); Cd36 (Rn02115479_g1); Arg1 (Rn00691090_m1); Cd86 (Rn00571654_m1); Tgfb1 (Rn00572010_m1); Mrc1 (Rn01487342_m1); 18S (Hs99999901_s1). Relative gene expression was calculated by the 2^-ΔΔCT^ method. (N = 5 animals/group)

### Immunochemistry

Western blot, dot blot, and immunohistochemistry were used to test amylin accumulation in brain samples, as previously [5] described. Western blots and dot blots were done on whole brain protein homogenates (N = 7 rats/per group/per study). Brain tissue from two HIP rats and one WT rat was examined by immunohistochemistry. An anti-human amylin antibody (polyclonal, raised in rabbit, T4157; Bachem-Peninsula, Laboratories, San Carlos, CA; 1:1000) that recognizes both human and rat amylin [(the latter with higher avidity (37)] was the primary antibody in immunohistochemistry and western blot tests. Another anti-human amylin primary antibody used was E-5 (monoclonal, SC 377530, Santa Cruz Biotech, Inc.; 1:500). Paraffin embedded pancreatic tissue from HIP rats was the positive control for amylin deposition. In the immunohistochemistry tests for ED1, anti-rat CD68 antibody (ED1, MCA341R, AbD Serotec, Raleigh, NC; 1:1000) was used as primary antibody. Bound primary antibodies in immunohistochemistry were detected with a biotinylated goat anti-rabbit IgG (Vector) for amylin and biotinylated donkey anti-mouse IgG (Jackson ImmunoResearch, West Grove, PA) for ED1 as the secondary antibodies. The specificity of anti-amylin antibody in immunohistochemistry studies was tested by incubating sections only with the secondary antibody (Additional file 1: Figure S1). The primary antibodies used in western blots for cytokines are TNFα (monoclonal, raised in rabbit, D2D4, XP, Cell Signaling tech, Inc; 1:1000), IL-6 (polyclonal, raised in rabbit, ab6672, Abcam; 1:1000) and IL-10 (Polyclonal, raised in rabbit, GTX74114, GeneTex Int.; 1:5000) The secondary antibodies used in western blots were anti-rabbit (Goat antirabbit, 32460, Thermoscientific) and anti-mouse (NA931, GEHealthcare). Equal loading in western blot experiments was verified by re-probing with a monoclonal anti-β actin antibody (raised in mouse, clone BA3R, Thermo Scientific; 1:2000).

For western blot, brain tissue was homogenized in homogenization buffer containing 10 mM Tris–HCl, pH 7.4, 150 mmol/L NaCl, 0.1% sodium dodecyl sulfate, 1% TritonX-100, 1% sodium deoxycholate, 5 mmol/L EDTA, 1 mmol/L NaF, 1 mmol/L sodium orthovanadate and protease and phosphatase inhibitor cocktail (Calbiochem). Brain lysate was generated by centrifugation (12,000 × g) of brain homogenate and collection of the supernatant fraction.

To estimate the amylin content in insoluble fractions from brain protein homogenates, pellets from brain homogenates were treated with formic acid (overnight), freeze dried and then the resulting powders were re-suspended in guanidine hydrochloride (2 hours). Dot blot analysis was used to assess amylin levels in post-treatment samples versus pre-treatment samples.

To detect the presence of oligomerized amylin in the circulation, blood samples from HIP rats and WT rats were collected in EDTA-treated tubes and centrifuged at 3500 rpm for 15 minutes to remove cellular components and platelets. Both resulting supernatant (plasma) and remaining pellet were analyzed by western blot.

After standard western and dot blot assays, the signal intensity of each band or dot was analyzed in Image J. To verify specific staining of protein bands, samples were loaded onto a gel in duplicate and after blotting and blocking, the membrane was cut and one half was incubated with the anti-amylin antibody while the other half was incubated in the absence of the primary antibody. Both halves were then incubated with the secondary antibody and developed and imaged together (Additional file 2: Figure S2).

### Behavioral testing

The SmartCage^TM^ system (AfaSci, Inc., Redwood City, CA) was used for automated analysis of spontaneous activity as described previously [49]. Homecage activity variables (active/inactive time, travel distance, velocity, and rear-ups) were determined by photo-beam breaks and automatically analyzed using CageScore software (AfaSci, Inc; HIP rats vs. WT rats, N = 5 rats/group). Each rat was monitored in an ordinary homecage placed within the SmartCage^TM^ platform for 72 hours, including the initial 24 hours for accommodation/acclimation. After the initial 24 hours of acclimation, recordings were made for the next 48 hours.

### Rotarod assessment

The rotarod (AfaSci, Inc., Redwood City, CA) was used to assess the vestibulomotor performance of the HIP rats and WT rats. Two days prior the first rotarod test, each animal was allowed 5 minutes to accommodate to the rotarod (learn to stand on the rod). Animals were probed at a rod-rotating speed of 15 rpm. The speed of the rotarod was increased from 0 rpm to 15 rpm within 1 min. Each rat was tested on the rotarod a total of 4 times per day over 5 consecutive days. For each training day, each rat’s shortest rotarod performance was discarded. The remaining 3 rotarod times were averaged, and then a group average was calculated for each genotype (HIP rats vs. WT rats; 9 months of age; N = 5 rats/group).

### Novel object recognition

This recognition memory task was conducted in an open field arena with two different kinds of objects which are different in shape and appearance [50,51]. In the acclimatization period, the animals were allowed to explore an empty arena. Twenty-four hours after this period, the animals were reintroduced to this environment with two identical objects placed in opposite corners of the arena. Four hours later, the animals were exposed to the familiar object and a novel object placed at the same locations for 5 minutes, during which the time exploring each object was recorded. A minimum of 10 sec of exploration was required, a criteria met by all rats in these experiments. The recognition index (RI) is defined as the ratio of time spent exploring the novel object over the total time spent exploring both familiar and novel objects. N = 11 pre-diabetic HIP rats (~9 months of age), N = 16 diabetic HIP rats (10–12 months of age) and N = 10 WT rats (~9 months of age) were used in this study. To assess possible regression in recognition memory with the development of disease, N = 3 pre-diabetic HIP rats were re-tested at a later time (~8 weeks), after they developed full-blown T2D (i.e., blood glucose >200 mg/dl). During the second NOR test, the above protocol was repeated using new objects for both the ‘familiar’ and the ‘novel’ object.

## Abbreviations

T2D: Type-2 diabetes; AD: Alzheimer’s disease; SD: Sprague–Dawley; qRT-PCR: Quantitative real-time PCR; WT: Wild-type; RI: Recognition index; NOR: Novel object recognition; ZDF: Zucker Diabetic Fatty; HIP: Human islet amyloid polypeptide.

## Competing interest

The authors declare that they have no competing interest.

## Authors’ contributions

SS performed animal studies. SS performed immunochemistry studies. ABB performed microglia gene expression measurements, analyzed data and reviewed the manuscript. JMB performed the NOR test. CP performed the home cage activity and rotarod tests. XSX analyzed and interpreted the data from home cage activity and rotarod tests, and reviewed the manuscript. KES analyzed and interpreted the data from the NOR test and reviewed the manuscript. LJVE analyzed and interpreted microglia gene expression data, and reviewed the manuscript. FD designed the study, analyzed data and wrote the manuscript. All authors read and approved the final manuscript.

## Supplementary Material

Additional file 1: Figure S1 The specificity of anti-amylin antibody in immunohistochemistry studies was tested by incubating sections only with the secondary antibody. Click here for file

Additional file 2: Figure S2 Matched pancreas and brain supernatant samples and plasma from the same HIP rats (N=2) were investigated for the presence of oligomerized amylin with the T-4157 anti-amylin antibody (left panel). To verify specific staining of protein bands, samples were loaded onto a gel in duplicate and after blotting and blocking, the membrane was cut and one half was incubated with the anti-amylin antibody while the other half was incubated in the absence of the primary antibody. Both halves were then incubated with the secondary antibody and developed and imaged together (right panel). Click here for file
