# Supplementary material for: Neuroinflammation and neurologic deficits in diabetes linked to brain accumulation of amylin
Source: Mol Neurodegener. 2014 Aug 22;9:30. doi: 10.1186/1750-1326-9-30 (PMC4144699; doi:10.1186/1750-1326-9-30)
Supplement: Additional file 1: Figure S1 — The specificity of anti-amylin antibody in immunohistochemistry studies was tested by incubating sections only with the secondary antibody. [file 1750-1326-9-30-S1.pdf]

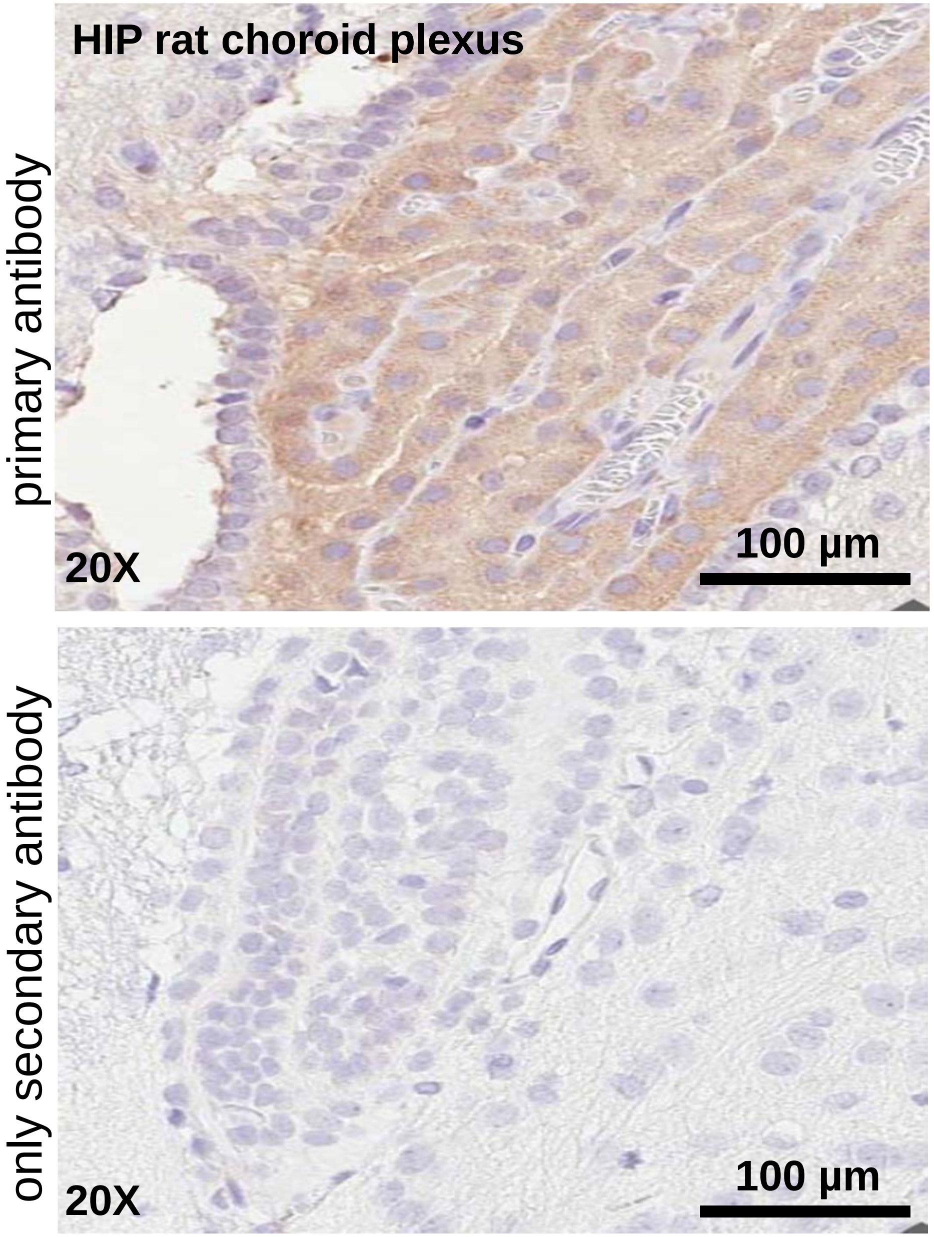

The specificity of anti-amylin antibody in immunohistochemistry studies was tested by incubating sections only with the secondary antibody.

Fig S1
