# Supplementary material for: Neuroinflammation and neurologic deficits in diabetes linked to brain accumulation of amylin
Source: Mol Neurodegener. 2014 Aug 22;9:30. doi: 10.1186/1750-1326-9-30 (PMC4144699; doi:10.1186/1750-1326-9-30)
Supplement: Additional file 2: Figure S2 — Matched pancreas and brain supernatant samples and plasma from the same HIP rats (N=2) were investigated for the presence of oligomerized amylin with the T-4157 anti-amylin antibody (left panel). To verify specific staining of protein bands, samples were loaded onto a gel in duplicate and after blotting and blocking, the membrane was cut and one half was incubated with the anti-amylin antibody while the other half was incubated in the absence of the primary antibody. Both halves were then incubated with the secondary antibody and developed and imaged together (right panel). [file 1750-1326-9-30-S2.pdf]

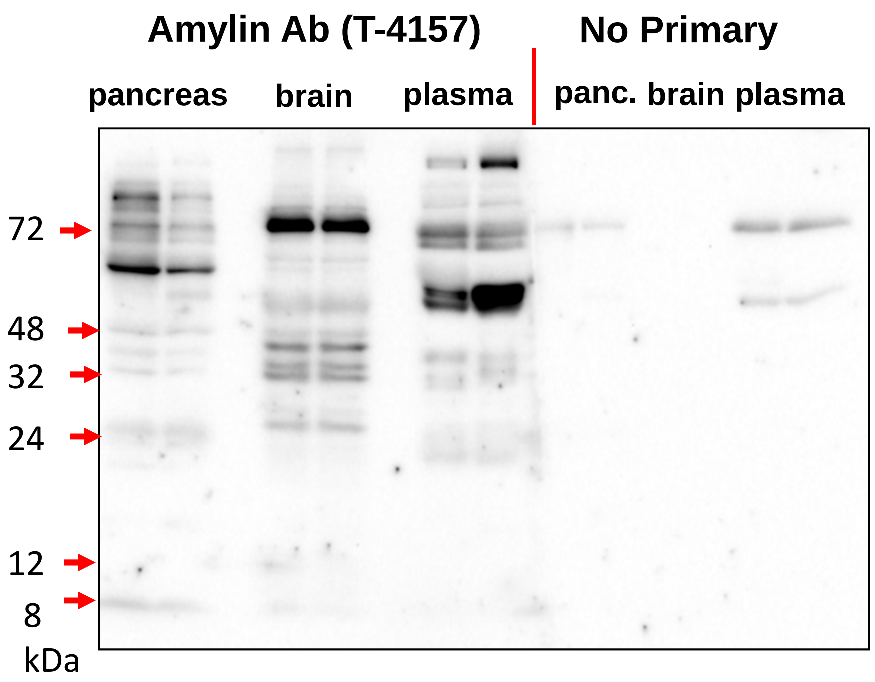

Matched pancreas and brain supernatant samples and plasma from the same HIP rats (N=2) were investigated for the presence of oligomerized amylin with the T-4157 anti-amylin antibody (left panel).

To verify specific staining of protein bands, samples were loaded onto a gel in duplicate and after blotting and blocking, the membrane was cut and one half was incubated with the anti-amylin antibody while the other half was incubated in the absence of the primary antibody. Both halves were then incubated with the secondary antibody and developed and imaged together (right panel).

Fig S2
